# Twist1 Directly Regulates Genes That Promote Cell Proliferation and Migration in Developing Heart Valves

**DOI:** 10.1371/journal.pone.0029758

**Published:** 2011-12-29

**Authors:** Mary P. Lee, Katherine E. Yutzey

**Affiliations:** Division of Molecular Cardiovascular Biology, Cincinnati Children's Hospital Medical Center, Cincinnati, Ohio, United States of America; Medical College of Wisconsin, United States of America

## Abstract

Twist1, a basic helix-loop-helix transcription factor, is expressed in mesenchymal precursor populations during embryogenesis and in metastatic cancer cells. In the developing heart, *Twist1* is highly expressed in endocardial cushion (ECC) valve mesenchymal cells and is down regulated during valve differentiation and remodeling. Previous studies demonstrated that Twist1 promotes cell proliferation, migration, and expression of primitive extracellular matrix (ECM) molecules in ECC mesenchymal cells. Furthermore, Twist1 expression is induced in human pediatric and adult diseased heart valves. However, the Twist1 downstream target genes that mediate increased cell proliferation and migration during early heart valve development remain largely unknown. Candidate gene and global gene profiling approaches were used to identify transcriptional targets of Twist1 during heart valve development. Candidate target genes were analyzed for evolutionarily conserved regions (ECRs) containing E-box consensus sequences that are potential Twist1 binding sites. ECRs containing conserved E-box sequences were identified for Twist1 responsive genes *Tbx20*, *Cdh11*, *Sema3C*, *Rab39b*, and *Gadd45a*. Twist1 binding to these sequences *in vivo* was determined by chromatin immunoprecipitation (ChIP) assays, and binding was detected in ECCs but not late stage remodeling valves. In addition identified Twist1 target genes are highly expressed in ECCs and have reduced expression during heart valve remodeling *in vivo*, which is consistent with the expression pattern of Twist1. Together these analyses identify multiple new genes involved in cell proliferation and migration that are differentially expressed in the developing heart valves, are responsive to Twist1 transcriptional function, and contain Twist1-responsive regulatory sequences.

## Introduction

The highly conserved basic helix-loop-helix (bHLH) transcription factor Twist1 was first identified in Drosophila as a critical regulator of mesoderm formation and specification [1]. In mouse and avian embryos, Twist1 functions in mesenchymal precursors of the developing pharyngeal arches, limb, cranial sutures, and heart valve endocardial cushions (ECC) [2]–[4]. Within these cell populations Twist1 promotes cell proliferation, migration, and expression of primitive extracellular matrix (ECM), thus promoting an undifferentiated state. In humans, highly metastatic and chemotherapeutic resistant cancers including breast, glioma, prostate, melanoma, and neuroblastoma express high levels of TWIST1 [5]. TWIST1 expression is also upregulated in human diseased aortic valves that have increased expression of mesenchymal markers of valve progenitor cells [4], [5]. The correlation of Twist1 expression with increased cell proliferation and migration of cancer cells, and also in diseased heart valves, is likely to be related to its functions in embryonic mesenchymal populations, including ECC mesenchymal cells. However, the underlying mechanisms by which Twist1 promotes proliferation and migration of mesenchymal cells during heart valve development and disease are largely unknown.

Mesenchymal valve progenitor cells of the ECCs are highly proliferative, migratory, and express ECM genes that encode the relatively unstructured and open matrix of the ECCs. As heart valve development progresses the valve progenitor cells begin to differentiate, which is marked by decreased proliferation, decreased migration, and expression of genes that encode the complex stratified ECM of the mature valves [6]. Within the mesenchymal cell population several factors, including Twist1, that promote cell proliferation and migration have been identified through both *in vivo* and *in vitro* studies [4], [7]–[10]. Previous gene expression profiling identified *Twist1* as the most differentially expressed gene during heart valve development with preferential expression in early ECC mesenchymal cells at embryonic day (E)12.5 and decreased expression in remodeling valve leaflets at E17.5 in mice [10]. In chick ECC explants, Twist1 promotes cell proliferation and migration consistent with a role in maintaining mesenchymal cells in an undifferentiated state [8]. There is limited information on the Twist1 target genes that mediate increased cell proliferation, migration, and primitive ECM gene expression. Expression of *Tbx20*, *Periostin*, *MMP13*, and *Cdh11* are responsive to Twist1 expression in ECCs, but it is not known whether they are direct or indirect transcriptional targets in the ECC mesenchymal cells [8]. Although Twist1 regulates cell proliferation and migration during expansion of the ECC mesenchymal cell population, the direct molecular mechanisms by which this occurs remain largely unknown.

Twist1 regulates gene expression primarily as a transcriptional activator through binding as a homodimer or heterodimer to the E-box DNA consensus sequence, CANNTG [11]. Twist1 forms homodimers (Twist1-Twist1) or heterodimers with other bHLH transcription factors, such as ubiquitously expressed E-proteins (E12/E47) [11], [12]. Previously identified Twist1 transcriptional targets, including *Periostin*, *N-cadherin*, *Collagen2a1*, and *Zyxin*, regulate adhesion-migration and ECM in various cell types [4], [13]–[15]. However, these targets do not account for all Twist1 function in ECC mesenchymal cells. Since Twist1 is highly expressed during heart valve development and disease, identifying the direct transcriptional targets will aid in understanding the mechanisms through which Twist1 promotes cell proliferation, migration, and primitive ECM gene expression.

We used a combination of approaches to identify direct transcriptional targets of Twist1 in ECC mesenchymal cells. Evolutionarily conserved regions (ECR) containing E-box consensus sequences were identified in chicken *Tbx20* and *Cdh11* genes, which were determined to be responsive to Twist1 in chick ECC studies [8]. Additionally, microarray gene expression profiling was performed on mouse preosteoblast cells (MC3T3-E1) transfected with Twist1 siRNA to identify additional candidate target genes containing ECRs. MC3T3-E1 cells express high levels of Twist1 and share significant gene expression with developing heart valves, thus facilitating Twist1 target gene identification [10]. Differential expression of candidate Twist1 target genes, including *Sema3C*, *Rab39b*, and *Gadd45a*, in developing heart valves in a pattern similar to Twist1 was confirmed *in vivo* in mice, and Twist1-responsive regulatory elements were identified. Furthermore, binding of Twist1 to candidate ECRs was confirmed in mouse embryonic heart valves *in vivo*. Each of the identified target genes has known functions in cell proliferation and migration, consistent with a role in expansion of ECC mesenchymal cells during heart valve development downstream of Twist1.

## Materials and Methods

### Ethics statement

All experiments with animals were carried out with experimental protocols and procedures reviewed and approved by the Cincinnati Children's Hospital Medical Center Biosafety Committee and Institutional Animal Care and Use Committee, protocol numbers 9D01009 and 0B08062.

### Genomic sequence analysis for ECRs

ECRs containing bHLH protein binding E-box consensus sequences (CANNTG) were identified using a combination of rVista2.0/ECR browser (http://rvista.dcode.org/ [16]), oPOSSUM (http://www.cisreg.ca/oPOSSUM/) [17], Trafac [18], and DiRe (Distant Regulatory elements of co-regulated genes, http://dire.dcode.org/ [19]) genome-wide analyses. ECR alignments were generated for homologous sequences based on sequence conservation in multiple species as identified by rVista analysis (Figure S1, Figure S2, Figure S3, Figure S4). *Tbx20boxA* (NW_001471633.1, bps 46990932 to 46991520 for luciferase assays and NW_001030907.1, bps 18507205 to 18507355 for ChIP) and *Cdh11-Intron1* (NW_001471435.1, bps 4202820 to 4203373 for luciferase assays and NW_001030904.1, bps 30738937 to 30739030 for ChIP) ECRs were identified using rVista2.0/ECR browser with chicken as the base genome. *Sema3C-Intron1* (NW_001030784.1, bps 1843993 to 1844225), *Gadd45a-promoter* (NW_001030811.1, bps 8955961 to 8956176) and *Rab39b-3′UTR* (NW_001035174.1, bps 660467 to 660626) ECRs were identified through a combination of rVista2.0/ECR browser, Trafac, DiRE, and oPOSSUM analyses with mouse as a base genome.

### Plasmids, transfections, and dual luciferase assay

Chicken *Tbx20boxA* and *Cdh11-Intron1* ECRs were amplified from genomic DNA isolated from white leghorn chicken embryos at E4.5 (Charles River, CT). 1 µg of chicken genomic DNA was used for PCR with the following primer sets and annealing temperatures: *Tbx20boxA* (5′- TAC GAG GGG GCT GTG AGG TCT -3′ and 5′- GCA AAG CAA GCA ATC GTG AA -3′, 55°C, 32 cycles) and *Cdh11-Intron1* (5′- GGT TGG GGT TGT TTA GGG TTT C -3′ and 5′- AGC CAT GTC TTC AGT GTC GTT TTA -3′, 56°C, 28 cycles). Mouse *Sema3CIntron1*, *Gadd45a-promoter*, and *Rab39b-3′UTR* ECRs were amplified from mouse genomic DNA isolated from cultured MC3T3-E1 cells (ATCC, CRL-2593) [20] with the following primer sets and annealing temperatures: *Sema3CIntron1* (5′- GGA AAG TCA CCC ATA AAA ATC AA -3′ and 5′- TAA ACA CAG CAT GCA ATC TCA AA -3′, 54°C, 35 cycles), *Rab39b-3′UTR* (5′- CTG GAA TAT AAG ACA ATC -3′ and 5′-CTG CAA TAA GTG GGT TTT -3′, 55°C, 30 cycles), and *Gadd45a-promoter* (5′- GCT GAA TCA TGA AGC TGT AAC TG -3′ and 5′- GGT TCA GGC AAT GCT TTT GT -3′, 55°C, 30 cycles). Amplified DNA was blunt ended with the Quick Blunting Kit (New England Biolabs, NEB) and DNA sequences ligated into the firefly luciferase vector, pGL3-promoter (pGL3p) linearized with SalI (NEB) and dephosphorylated with calf intestinal alkaline phosphatase (NEB) according to manufacturer's instructions. The ECR fragment sequence and orientation within pGL3p was verified through DNA sequencing.

Human embryonic kidney (HEK) 293 cells (ATCC, CRL-1573) were transfected using FUGENE6 Transfection Reagent (Roche) according to the manufacturer's protocol and as previously described [4]. HEK 293 cells were grown as previously described on 60 mm plates (Fisher) for 24 hours to 40–50% confluency [4]. Cultures were co-transfected with 0.5 µg of the firefly luciferase constructs containing the ECRs of interest and either 0.1 µg of control empty vectors (pcDNA3.1 or pEMSV) or 0.1 µg of expression plasmids pcDNA-Twist1 [3] (gift from Dr. Anthony Firulli, Indiana University School of Medicine) and/or pEMSV-E12 (gift from Dr. Jeffery Molkentin, Cincinnati Children's Hospital Medical Center, CCHMC). All samples were co-transfected with 0.01 µg renilla luciferase (pRL-TK, promega) reporter plasmid for normalization of transfection efficiency [4]. Transfected cells were incubated for 48 hours at 37°C with 5% C0_2_, then washed (1XPBS), and lysed (1X Passive Lysis Buffer, Promega) according to the manufacturer's protocol [4]. Cells were subjected to one freeze thaw cycle to facilitate lysis, then thawed and centrifuged at 13,000 rpm for 1 minute to collect cell debris. Dual-luciferase (Promega) assays were performed according to the manufacturer's protocol with 20 µl of cell lysate supernatant used to evaluate firefly luciferase and renilla luciferase levels using a single sample reader Monolight luminometer (BD Pharmingen) [4], [21]. For all samples, firefly luciferase values were normalized relative to renilla luciferase values. Average fold change and standard error of the mean (SEM) were calculated from at least 3 independent co-transfection experiments performed in triplicate. Statistical significance was determined by Student's *t*-test (p≤0.05).

### Site-directed mutagenesis

Site-directed mutagenesis of *Tbx20boxA*, *Cdh11-Intron1*, and *Sema3C-Intron1* E-box consensus sequences was performed on each ECR within pGL3p vector using QuickChange Site-Directed Mutagenesis Kit (Stratagene) according to the manufacturer's protocol [4], [21]. E-box consensus sequences (CANNTG) were mutated at 3 nucleotides (ATNNAG) [22]. The following primers and annealing temperatures were used for E-box mutagenesis: *Tbx20boxAMut* (5′- GCC TGT CTA ATT AGT ATT AAG AAC GGA GGG C -3′ and 5′- CGG ACA GAT TAA TCA TAA TTC TTG CCT CCC G -3′, 55°C, 12 cycles), *Cdh11-Intron1Mut* (5′- GGT ACA ATG AAA GAA TTT AGT AAA TGA AGC AGA TAA GCC C-3′ and 5′- GGG CTT ATC TGC TTC ATT TAC AAA TGT CTT TCA TTG TAC C-3′, 62°C, 16 cycles), *Sema3C-Intron1Mut* (5- AAA CAT CTC TAG GGT CTC CTC ATT CAG TGT GGT AGA GGC AGA G -3′ and 5′- CTC TGC CTC TAC CAC ACT GAA TGA GGA GAC CCT AGA GAT GTT T -3′, 55°C, 12 cycles). The predicted nucleotide changes were verified through DNA sequencing for each reporter plasmid. Mutated constructs (*Tbx20boxA-Mut*, *Cdh11-Intron1Mut*, and *Sema3C-Intron1Mut*) were then used for dual-luciferase assays as described above.

### Chromatin immunoprecipitation (ChIP)

*In vivo* binding of Twist1 protein to DNA was detected by ChIP assay in mouse E12.5 ECCs and E17.5 remodeling atrioventricular (AV) valves. Litters were generated from timed matings of FVBN wild-type (Taconic) mice where the presence of a copulation plug was considered E0.5. Pregnant females were sacrificed with CO_2_ inhalation and embryos isolated. Tissue was dissected from 10–12 atrioventricular canal (AVC) E12.5 ECCs or E17.5 AV valves [10]. Dissected ECCs and AV valves were placed in DMEM medium (Invitrogen) supplemented with 10% FBS (HyClone) and 1% penicillin-streptomycin (pen-strep, Invitrogen). Tissue was treated with a final concentration of 3.7% formaldehyde (Sigma) for 10 minutes to cross-link protein/chromatin complexes followed by lysis by sonication (Virsonic 60, Virtis) 2 times for 10 seconds with a 5-minute refractory period and an output of 5. ChIP was then performed according to manufacturer's protocol (EZChIP, Millipore) modified by use of protein A-agarose beads (Millipore) [4], [23], [24]. Immunoprecipitation (IP) was performed with a Twist1 specific antibody (Sigma T6451, 5 µg) or control normal rabbit IgG (Cell Signaling, 5 µg). Eluted DNA from ChIP samples was evaluated by quantitative polymerase chain reaction (qPCR) relative to normal rabbit IgG control [25]. qPCR amplification reactions were performed with initial denaturation of 94°C for 3 min, 25 cycles of 94°C for 20 s, annealing temperature dependent based upon the primer set for 30 s, 72°C for 30 s, and final extension at 72°C for 2 min. PCR amplification was performed using the following primers and annealing temperatures: *Tbx20boxAChIP* (5′- AAG CAT GGA TTG TTG AGG AAG T -3′ and 5′- CTA AGA GAA AGC AGG CTA CAT AAG -3′, 55°C), *Cdh11Intron1E-box1* (5′- TGC GAC TGA TAA GAC TGC CAT TG -3′ and 5′- GAA AGG CCC ATT GTG CTG CTA C -3′, 55°C), *Cdh11Intron1E-box2ChIP* (5′- GAA AGG CCC ATT GTG CTG CTA C -3′ and 5′- CTG CCT GAG CCT CCT GAC TG -3′, 55°C), *Sema3CIntron1ChIP* (5′- GGA AAG TCA CCC ATA AAA ATC AA -3′ and 5′- TAA ACA CAG CAT GCA ATC TCA AA -3′, 56°C), *Rab39b3′UTRChIP* (5′- CTG GAA TAT AAG ACA ATC -3′ and 5′-CTG CAA TAA GTG GGT TTT -3′, 45°C), and *Gadd45a-promoterChIP* (5′- GCT GAA TCA TGA AGC TGT AAC TG -3′ and 5′- GGT TCA GGC AAT GCT TTT GT -3′, 55°C). SEM and fold enrichment were calculated relative to IgG control, set to 1, from 3 independent ChIP experiments performed in triplicate [25]. Statistical significance was determined by Student's *t*-test (p≤0.05).

### siRNA knockdown

Double stranded siRNAs with a 3′ dT overhang specific to mouse *Twist1* were designed using Block-it RNAi designer (Invitrogen) [8]. To efficiently knockdown *Twist1*, MC3T3-E1 cells were transfected with a pool of 3 double stranded siRNAs (total concentration of 200 nM) for each experiment. The siRNA sequences were: mTwist1-1 (GCAAGAUUCAGACCCUCAA and UUGAGGGUCUGAAUCUUGC), mTwist1-2 (GGUGUCUAAAUGCAUUCAU and AUGAAUGCAUUUAGACACC), and mTwist1-3 (CCGCCAGAGAUUGUAGCAU and AUGACAUCUAGGUCUCCGG). Scrambled siRNA (AAACAUGCCUAGAGAGAGC and GCUCUCUCUAGGCAUGUUU) was used as a control. MC3T3-E1 cells were cultured in 60 mm dishes for 24 hours in MEM-alpha medium (Invitrogen), 10% FBS, and 1% pen-strep and transfected at 50–60% confluency. Cells were then washed 3 times with 1XPBS and incubated with 1 ml OPTI-MEM (Invitrogen) during preparation of siRNA mixture for transfection [8]. Lipofectamine 2000, OPTI-MEM, and 200 nM siRNA were mixed according to manufacturer's protocol and as previously described [8]. The siRNA mixture (Lipofectamine 2000/OPTI-MEM/siRNA) was added to cells in OPTI-MEM and incubated for 4–6 hours. The siRNA mixture/OPTI-MEM was then removed and cells were incubated in MEM-alpha media/10%FBS/1%pen-strep for an additional 48 hours. RNA was isolated from all samples using Trizol Reagent (Invitrogen) and additional purification was performed using RNeasy Mini Kit (Qiagen) [10]. siRNA transfection efficiency in MC3T3-E1 cells was evaluated utilizing Block-it Fluorescent Oligo (Invitrogen) reagent transfected with Lipofectamine 2000 and imaged as previously reported [7]. Percent transfection was calculated by comparing the total number of fluorescently labeled cells to the total nuclei in 10 fields of cells from 3 experiments. Transfection efficiency was ∼80% for three independent experiments (n = 3). siRNA knockdown of Twist1 was approximately 80% as assessed by qPCR.

### Affymetrix microarray hybridization and gene expression analysis

Total purified mRNA isolated from Twist1 siRNA (siTwist1) or Scrambled siRNA (siScr) transfected MC3T3-E1 cells was submitted in biological triplicate to Cincinnati Children's Hospital Medical Center Affymetrix Microarray core for gene expression analysis [10]. RNA integrity of all six samples was confirmed using an Agilent 2100 Bioanalyzer (Agilent Technologies) and RNA 6000 Nano Assay [10]. Double stranded cDNA was generated from 400 ng of each sample (3-siScrambled and 3-siTwist1) using the TargetAMP1-Round Aminoallyl-RNA Amplification kit (Epicenter). Biotin-labeled cRNA was synthesized with the IVT Labeling Kit (Affymetrix) then chemically fragmented and hybridized to Mouse Genome 430 2.0 Array (Affymetrix) using standard protocols. Arrays were washed and stained with Fluidics Station 450 (Affymetrix), scanned with GeneChip Scanner 3000 (Affymetrix), with the scanned gene expression data exported as .CEL files. Data were loaded into GeneSpring Gx 7.3 software (Agilent Technologies) and quantile normalization was performed with robust multichip average (RMA) analysis [10]. Statistical analysis (ANOVA) identified 5637 probe sets with significantly differential gene expression (p≤0.05) 5637 and 65 genes with ≥2.0 fold decreased gene expression. The complete MIAME compliant data set is can be accessed through the GEO database with the accession number GSE30953.

### RNA isolation and quantitative RT-PCR

Tissue from 10–12 E12.5 AVC ECCs and E17.5 AV valves of FVBN wild-type mouse embryos was isolated with tungsten needles in 1XPBS then placed in Trizol Reagent for RNA isolation as previously described [4], [10]. RNA was isolated from MC3T3-E1 cells at 80–90% confluency cultured in a 100 mm tissue culture dish (Fisher) using Trizol Reagent [10], [26]. cDNA was generated using Superscript cDNA kit (Invitrogen) with 800 ng of RNA. qPCR was performed as previously described [7]. For each primer set, standard curves generated with cDNA from MC3T3 cells were used to determine cycle threshold, and all samples were normalized for input based on expression levels of housekeeping control gene, L7 [10]. The following primer sets and annealing temperatures were used for qPCR analysis of murine candidate target genes: *Rab39b* (5′- GGC TCG ATC TCC ACC AAA CG -3′ and 5′- ACC AGT TCC GGC TCA TTG TG -3′, 62.5°C), *Tubb3* (5′- TAG ACC CCA GCG GCA ACT AT -3′ and 5′- GTT CCA GGT TCC AAG TCC ACC -3′, 62.5°C), *Gadd45a* (5′- TGC TGC TAC TGG AGA ACG AC-3′ and 5′- CGA CTT TCC CGG CAA AAA CAA A -3′, 62.5°C), *Serpinb9b* (5′- AAG GAG TCC TGT TTT CGC TTC -3′ and 5′- CTG AGT CAT CTG CCA ACA ACT -3′, 60.0°C), *Pa2g4* (5′- CAG CAG GAG CAA ACT ATC GC -3′ and 5′- GGC ATC ACC TTT CTC ACA CAA G -3′, 61.0°C), *Trib3* (5′- TGC AGG AAG AAA CCG TGG GAG -3′ and 5′- CTC GTT TTA GGA CTG GAC ACT TG -3′, 61.0°C), *Nras* (5′- ACT GAG TAC AAA CTG GTG GTG G -3′ and 5′- TCG GTA AGA ATC CTC TAT GGT GG -3′, 61.0°C), and *Sema3C* (5′- ACA GCA GGA AAA GCA GAA ACA GGA -3′ and 5′- CAG CAG CCG ACA CAT CTT ACA ATC -3′, 59.0°C). Primer specificity was determined by DNA sequencing following ligation of amplified fragment into pGEM-T (Promega) vector. Previously validated primers were used for amplification of *Twist1*, *Osteonectin*, *Col2a1*, and *Col5a1* [10], [27]. Average fold change of qPCR values for E17.5 AV valves was compared to E12.5 ECCs set to 1.0, then the SEM was calculated from 3 independent experiments performed in triplicate. Statistical significance was determined by Student's *t*-test (p≤0.05).

### Probe generation and In situ hybridization (ISH)

The following primer sequences and annealing temperatures were used to generate PCR fragments for anti-sense riboprobes for ISH: *Tubb3* (5′- TCT GGC GCC TTT GGA CAC CTA TT -3′ and 5′- CAT GCG CCC ACG GAA GAC AGT -3′, 64°C), *Rab39b* (5′- GCG AGC GCA GCA TCC ATC C -3′ and 5′- CTT CAC CCC TCC CCA ACC CTC CTG -3′, 54°C), and *Serpinb9b* (5′- AGT CCA GGC AAT GCA TAA ACA GC -3′ and 5′- GGG CCA CCA CCT AAG CAG AGA -3′, 56.8°C). All sequences were amplified by RT-PCR of MC3T3-E1 cell cDNA. *Gadd45a* primers were previously described, and the *Sema3C* plasmid (*Sema3CpSport6*) was a kind gift from Dr. Yutaka Yoshida, CCHMC [28]. *Twist1* ISH probe was a kind gift from Dr. James Martin, Texas A&M Health Sciences Center [29]. All primers PCR fragments were ligated into pGEM-T vector (Promega) using Rapid T4 DNA Ligase (Roche, 11635) [4]. Amplification of predicted sequences by each primer set was confirmed by DNA sequencing. Digoxigenin (DIG)-labeled ISH antisense riboprobes were generated as previously described with the following modifications [30]. The *Sema3CpSport6* plasmid was linearized with SalI and probe synthesized with T7 polymerase. *Serpinb9b* and *Gadd45a* plasmids were linearized with NotI and probes synthesized with SP6 polymerase. *Tubb3* and *Rab39b* plasmids were linearized with NcoI and probes synthesized with SP6 polymerase. The *Twist1* plasmid was linearized with XbaI and probe synthesized with T3 polymerase.

FVBN wild-type mouse E12.5 whole embryos and E17.5 hearts were isolated and fixed in 4% paraformaldehyde (PFA, Electron Microscopy Sciences), then embedded in paraffin wax as previously described [31]. Paraffin-embedded samples were sectioned at a thickness of 14 µm. ISH was performed as previously described with the following modifications [7]. Sections were treated with 20 µg/ml proteinase K/PBS for 10 minutes at 37°C. Hybridization was carried out as previously described. For all ISH experiments, color reactions with tetrazolium/5-bromo-4-chloro-3-indolyl phosphate (NBT/BCIP, Roche) on E12.5 embryos and E17.5 heart sections were stopped at the same time for each probe. Development of color reactions ranged from 4 to 16 hours.

## Results

### Twist1 binds and promotes gene expression from an ECR located upstream of cTbx20

**Figure 1:**
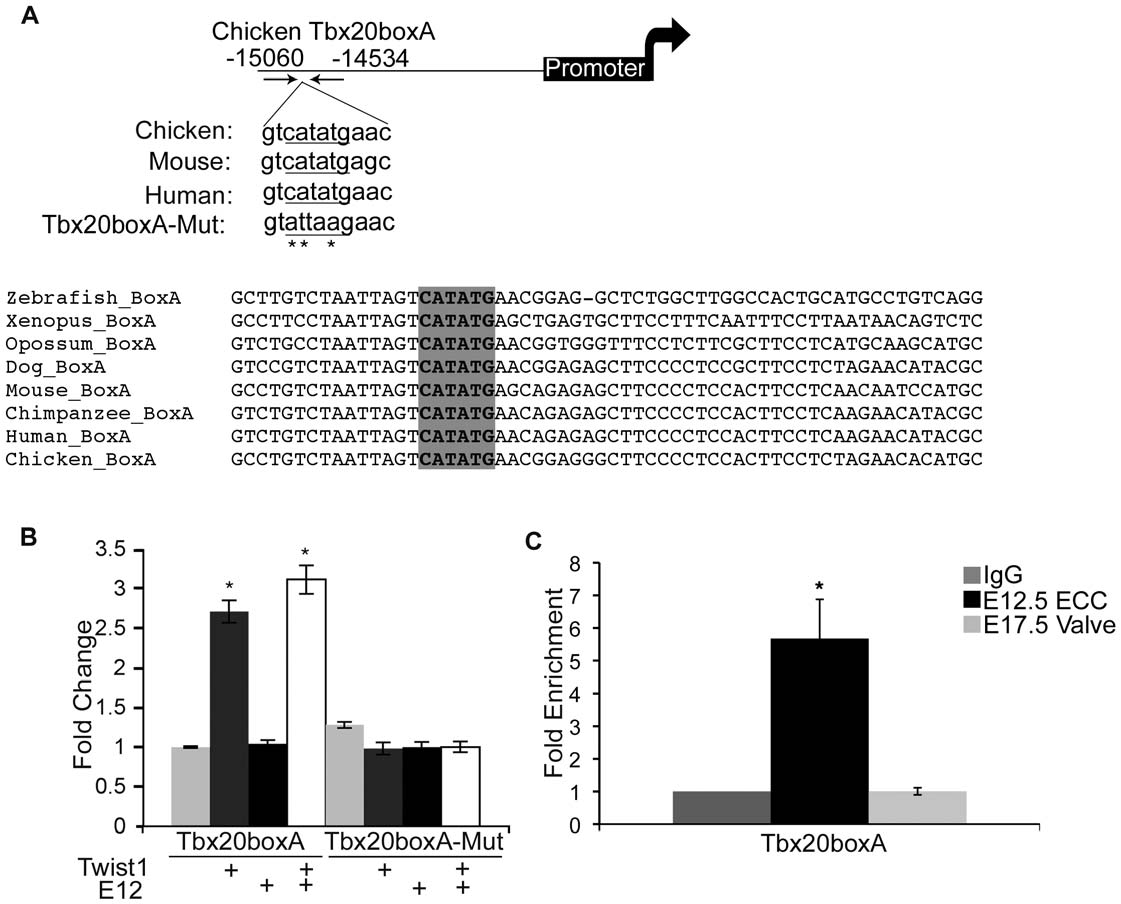
Twist1 binds and promotes gene expression from an ECR associated with *Tbx20 (Tbx20boxA)*. A. An E-box-containing ECR is located −15040 to −14862 base pairs (bps) from the chicken *Tbx20* transcriptional start site (+1). Cross-species genomic alignment of the E-box consensus sequence (shaded grey) and mutated sequence (mutated bps indicated by *) are indicated. B. *Tbx20boxA* or *Tbx20boxA-Mut* plasmids were co-transfected into HEK293 cells, with empty vector or with Twist1, E12, or Twist1 and E12 expression vectors followed by luciferase reporter assays. Fold change over the empty vector control set to 1 is shown with standard error of the mean (SEM). C. ChIP assays were performed with anti-Twist1 in mE12.5 ECCs and mE17.5 AV valves quantified by qPCR. Fold enrichment was evaluated by comparing anti-Twist1 IP of E12.5 ECCs or anti-Twist1 IP of E17.5 valves versus IgG (negative control) set to 1. Statistical significance was determined by Student's *t*-Test, p = ≤0.05 indicated by *. All experiments were performed in biological and technical triplicate. Histograms are a compilation of n = 3 experiments.

In ECCs *Tbx20* gene expression is responsive to Twist1, however, whether this relationship is direct or indirect has not been reported previously [8]. rVista 2.0 analysis was performed for *Tbx20* gene sequence alignment using chicken as the base genome in order to determine if *Tbx20* contains candidate DNA sequences directly regulated by Twist1. An ECR (conserved from human to zebrafish) and containing a conserved E-box consensus sequence was identified −15040 to −14862 base pairs (bps) upstream from the c*Tbx20* transcriptional start site (+1 site), which will be referred to as *Tbx20boxA* (Figure 1A). Additional ECRs were identified that are located further away from the +1 site of *Tbx20*. However, these ECRs are more than 20 Kb from the +1 site and do not contain E-box consensus sequences.

Twist1 responsiveness of the *Tbx20boxA* ECR was determined by co-transfection assays in HEK 293 cells. The *Tbx20boxA* sequence was linked to the minimal SV40 promoter of pGL3p, which contains a firefly luciferase reporter, to generate *Tbx20boxA/pGL3p*. Co-transfection of *Tbx20boxA/pGL3p* with a Twist1 expression plasmid results in approximately 2.75-fold increase in reporter gene activity compared to *Tbx20boxA/pGL3p* co-transfected with control empty vector (pCDNA, Figure 1B). Co-transfection of *Tbx20boxA/pGL3p* with E12 alone has no effect on reporter gene expression. However, co-transfection of *Tbx20boxA/pGL3p* with both Twist1 and E12 expression vectors results in approximately 3.25-fold increase in reporter gene activation. These experiments confirm that *Tbx20boxA* has enhancer activity when linked to a minimal SV40 promoter. To confirm that the E-box consensus sequence is essential for Twist1 to promote gene expression from *Tbx20boxA/pGL3p*, site-directed mutagenesis was performed to generate *Tbx20boxAMut/pGL3p* (CATATG to ATTAAG, Figure 1A). Co-transfection of *Tbx20boxAMut/pGL3p* with Twist1 alone, E12 alone, or Twist1 and E12 together confirms that the E-box consensus sequence is necessary for Twist1 to promote gene expression from *Tbx20boxA/pGL3p* (Figure 1A,B). ChIP assays were utilized to assess Twist1 direct binding to *Tbx20boxA in vivo* during murine early (E12.5 ECC) and late (E17.5 AV valve) valve development (Figure 1C). Immunoprecipitation with anti-Twist1 demonstrates that Twist1 is bound to the *Tbx20boxA* sequence in E12.5 ECCs evident by approximately 3.75-fold enrichment compared to IgG control. However, no enrichment in Twist1 binding to *Tbx20boxA* was observed in E17.5 AV valves in which *Twist1* gene expression is downregulated (Figure 1C). Thus Twist1 promotes gene expression from a novel enhancer associated with *Tbx20*, *Tbx20boxA*, in an E-box dependent manner in transfected cells. Furthermore, Twist1 directly binds to *Tbx20boxA* in ECC mesenchymal cells, but not remodeling heart valves, *in vivo*.

### Twist1 binds and promotes gene expression from an ECR in Intron1 of Cdh11

**Figure 2:**
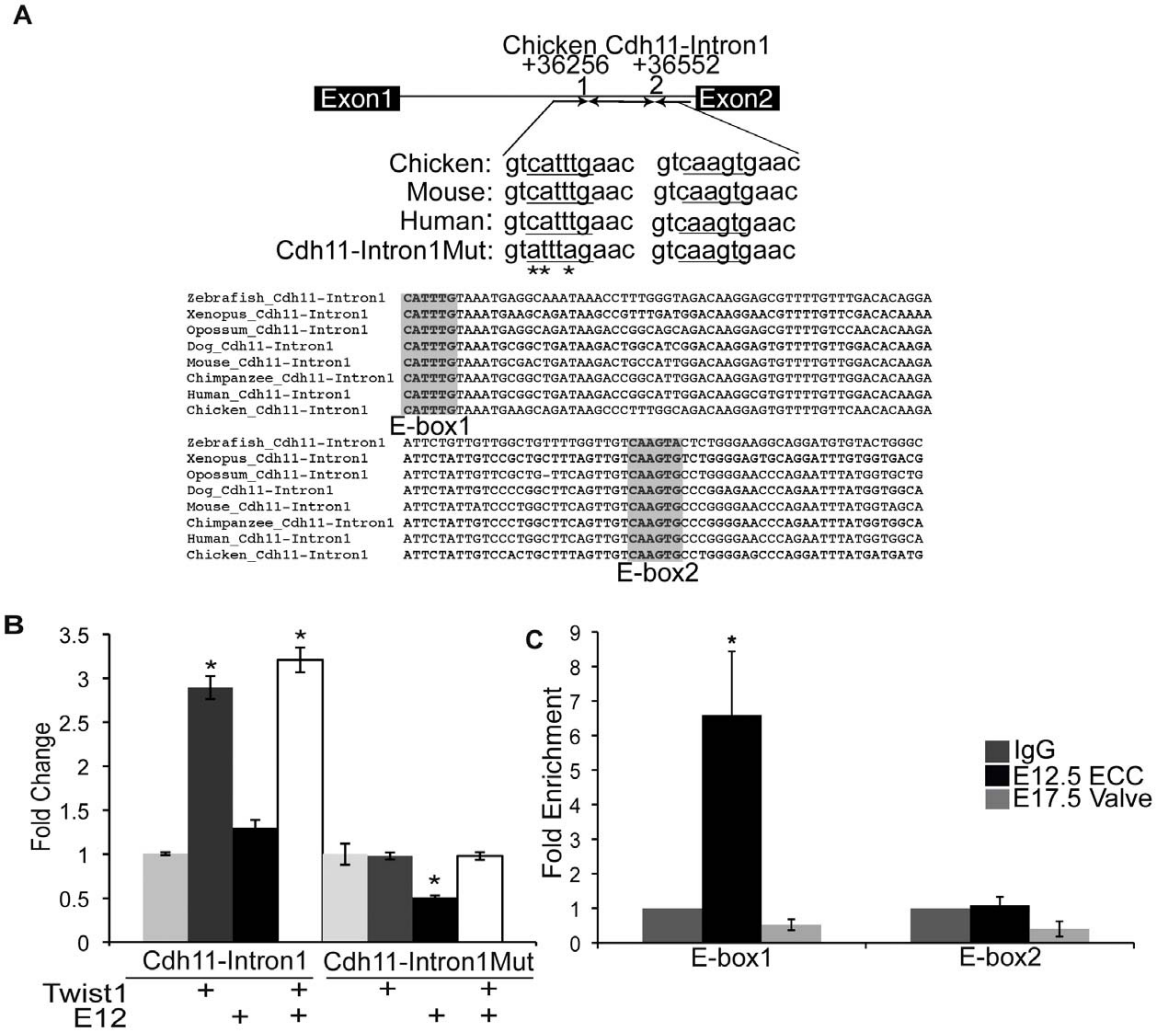
Twist1 binds and promotes gene expression from an ECR within Intron1 of *Cadherin-11 (Cdh11-Intron1)*. A. An E-box-containing ECR is located +36256 to +36552 bps from the chicken *Cdh11* transcriptional start site (+1). Cross-species genomic alignment of the E-box consensus sequence (shaded grey) and mutated sequence (mutated bps indicated by *) are indicated. B. *Cdh11-Intron1* or *Cdh11-Intron1Mut* plasmids were co-transfected into HEK293 cells with empty vector or with Twist1, E12, or Twist1 and E12 expression vectors and luciferase reporter assays performed. Fold change over the empty vector control set to 1 is shown with SEM. C. ChIP assays were performed with anti-Twist1 in mE12.5 ECCs and mE17.5 AV valves and quantified by qPCR. Fold enrichment was evaluated by comparing anti-Twist1 IP of mE12.5 ECCs or anti-Twist1 IP of mE17.5 valves versus IgG (negative control) set to 1. Statistical significance was determined by Student's *t*-Test, p = ≤0.05 indicated by *. All experiments were performed in biological and technical triplicate. Histograms are a compilation of n = 3 experiments.

The adhesion-migration molecule *Cdh11* (*OB-Cadherin*) is highly expressed in ECC mesenchymal cells [8]. Furthermore, in chick ECC cultures *Cdh11* expression is responsive to Twist1 expression. Genomic alignment and transcription factor binding site analysis was performed to identify ECRs containing E-box consensus sequences in the *Cdh11* gene using chicken as the base genome. An ECR (conserved in human to zebrafish) containing two conserved E-box consensus sequences was identified within the first intron of chicken *Cdh11 (Cdh11-Intron1*, +36256 to +36552 bps from the +1 site, Figure 2A). *Cdh11-Intron1* sequence was amplified from E4.5 chicken genomic DNA and linked to the minimal SV40 promoter of the pGL3p luciferase reporter plasmid. In co-transfection assays, Twist1 promotes gene expression from *Cdh11-Intron1*/pGL3p approximately 2.75-fold versus *Cdh11-Intron1*/pGL3p with empty vector, while co-transfection with E12 alone has no observed effect on reporter gene expression (Figure 2B). Co-transfection with Twist1 and E12 together results in activation of approximately 3.25-fold that is not statistically different from co-transfection with Twist1 alone. These data identify a novel Twist1-responsive enhancer within the first intron of *Cdh11*, *Cdh11-Intron1*.

Since two E-box consensus sequences are present within *Cdh11-Intron1*/pGL3p, Twist1 could bind and promote gene expression from either or both E-box consensus sites (E-box1 or E-box2, Figure 2A). To examine whether Twist1 preferentially activates E-box1 or E-box2, site-directed mutagenesis was performed on E-box1 (CATTTG to ATTTAG, *Cdh11-Intron1Mut/pGL3p*, Figure 2A). Loss of E-box1 in *Cdh11-Intron1Mut*/pGL3p eliminates reporter gene activity by Twist1, E12, or Twist1 with E12, indicating that Twist1 transactivation is dependent upon an intact E-box1 consensus sequence (Figure 2B).

To examine the ability of Twist1 to directly bind to the *Cdh11-Intron1* ECR in developing heart valves *in vivo*, ChIP was performed in mouse E12.5 ECCs and E17.5 AV valves (Figure 2C). Twist1 immunoprecipitation is enriched 6.5- to 8-fold for a region of *Cdh11-Intron1* that contains only E-box1, but no Twist1 binding is detected for a region of *Cdh11-Intron1* that contains only E-box2, relative to the IgG control. Therefore, Twist1 preferentially binds and promotes gene expression from the *Cdh11-Intron1* E-box1 sequence (Figure 2C). Twist1 immunoprecipitation is not enriched with *Cdh11-Intron1* regions that contain either E-box1 or E-box2 in E17.5 AV valves (Figure 2C). Together, these data indicate that Twist1 directly binds to *Cdh11-Intron1* in E12.5 ECC mesenchymal cells and promotes gene expression specifically through the E-box1 consensus site.

### Identification of Twist1 candidate genes by siRNA-mediated loss of function in MC3T3-E1 cells

**Figure 3:**
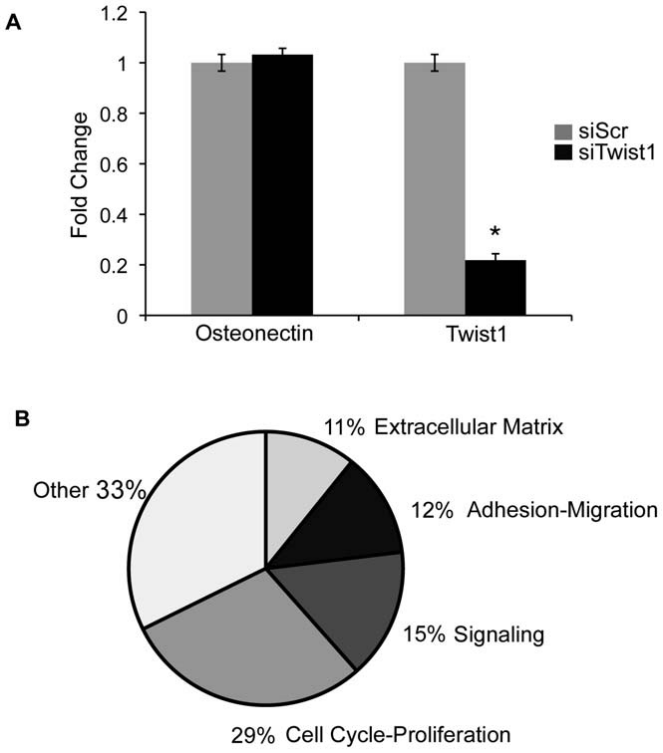
Knockdown of Twist1 results in decreased expression of genes associated with mesenchymal cell functions. A. qPCR of *Twist1* and *Osteonectin* expression confirms knockdown of *Twist1* in MC3T3-E1 pre-osteoblast cells transfected with siTwist1 versus siScr controls. Fold change relative to siScr transfected cells, set to 1, ± SEM is shown. Statistical significance was determined by Student's *t*-Test, p = ≤0.05 indicated by *. All experiments were performed in biological and technical triplicate. Histograms are a compilation of n = 3 experiments. B. Twist1 candidate target genes from gene expression profiling with ≥2.0 fold decreased grouped by functional classifications are represented in a pie graph.

In order to identify additional Twist1 target gene candidates, an siRNA-mediated knockdown and gene expression profiling approach was employed in transfected MC3T3-E1 preosteoblast cells. MC3T3-E1 cells are a preosteoblast cell line that expresses high levels of *Twist1* and has extensive shared gene expression with E12.5 ECCs [10], [20]. Therefore, MC3T3-E1 cells were chosen as an appropriate *in vitro* system for identification of Twist1 candidate target genes. MC3T3-E1 cells were transfected with a combination of 3 siRNAs specific to Twist1 (siTwist1) or scrambled control siRNA (siScr). Twist1 siRNA transfection resulted in approximately 80% *Twist1* mRNA knockdown verified through qPCR and loss of protein as determined by immunohistochemistry (Figure 3A and data not shown). RNA was collected from MC3T3-E1 cells transfected with siScr or siTwist1 and subjected to gene expression profiling using Affymetrix Microarray analysis. Gene expression data were normalized and prioritized according to p-value (p≤0.05) and fold change (≥2.0 fold) comparing MC3T3-E1 cells treated with either siTwist1 or siScr. A total of 65 genes were identified with decreased expression by at least 2-fold in cells transfected with siTwist1 versus siScr.

The 65 genes with ≥2-fold decreased expression with knockdown of Twist1 were subjected to PANTHER analysis for biological function classification. The majority of the differentially expressed genes are classified into biological functions that are consistent with known Twist1 functions during development and disease (Figure 3B, Table 1) including cell cycle-proliferation (29%), cell signaling (15%), adhesion-migration (12%), and extracellular matrix (11%). The remaining genes were classified into an “other” (33%) category, consisting of biological classifications such as ion transport, calcium binding, biotin metabolism, and chemotaxis. Thus Twist1 knockdown in MC3T3-E1 preosteoblast cells results in gene expression changes consistent with known Twist1 regulation of cell proliferation, adhesion-migration, signaling, and ECM gene expression.

**Table 1:** Fold change and functional classification of Twist1 candidate target genes with the most decreased gene expression in siTwist1 vs. siScr transfected MC3T3-E1 cells.

Adhesion-Migration			
Ref Seq	Gene ID	Gene Name	Fold Change
NM_013657	Sema3C	Semaphorin 3C	−4.95
NM_023279	Tubb3	Tubulin, beta 3	−4.27
NM_010717	Limk1	LIM-domain containing, protein kinase	−2.74
NM_145953	Cth	Cystathionase (cystathionine gamma-lyase)	−2.60
NM_010833	Msn	Moesin	−2.19
NM_008659	Myo1c	Myosin IC	−2.16
NM_001081053	Itga10	Integrin, alpha 10	−2.12
NM_053083	Loxl4	Lysyl oxidase-like 4	−2.06
NM_011693	Vcam1	Vascular cell adhesion molecule 1	−2.09
**Extracellular Matrix**			
NM_015734	Col5a1	Procollagen, type V, alpha 1	−2.99
NM_011434	Sod1	Superoxide dismutase 1, soluble	−2.42
NM_007729	Col11a1	Procollagen, type XI, alpha 1	−2.40
NM_025711	Aspn	Asporin	−2.30
NM_010917	Nid1	Nidogen 1	−2.21
NM_010721	Lmnb1	Lamin B1	−2.17
NM_008695	Nid2	Nidogen 2	−2.12
**Signaling Molecules**			
NM_011452	Serpinb9b	Serine (or cysteine) peptidase inhibitor, clade B, member 9b	−3.41
NM_175093	Trib3	Tribbles homolog 3 (Drosophila)	−2.95
NM_146162	Tmem119	Transmembrane protein 119	−2.54
NM_028744	Pi4k2b	Phosphatidylinositol 4-kinase type 2 beta	−2.53
NM_019681	Freq	Frequenin homolog (Drosophila)	−2.25
NM_024454	Rab21	RAB21, Member RAS oncogene family	−2.18
NM_009369	Tgfbi	Transforming growth factor, beta induced	−2.17
NM_021532	Dact1	Dapper homolog 1, antagonist of beta-catenin (xenopus)	−2.20
NM_008924	Prkar2a	Protein kinase, cAMP dependent regulatory, type II alpha	−2.12
NM_025954	Pgp	Phosphoglycolate phosphatase	−2.07
NM_008845	Pip4k2a	Phosphatidylinositol-5-phosphate 4-kinase, type II, alpha	−2.05
**Cell Cycle-Proliferation**			
NM_007836	Gadd45a	Growth arrest and DNA-damage-inducible 45 alpha	−4.13
NM_021288	Tyms	Thymidylate synthase	−3.91
NM_175122	Rab39b	RAB39B, Member RAS oncogene family	−3.64
NM_011119	Pa2g4	Proliferation-associated 2G4	−3.13
NM_010937	Nras	Neuroblastoma ras oncogene	−2.73
NM_010485	Elavl1	ELAV (Embryonic lethal, abnormal vision, Drosophila)-like 1 (Hu antigen R)	−2.62
NM_145953	Cth	Cystathionase (cystathionine gamma-lyase)	−2.60
NM_011641	Trp63	Transformation related protein 63	−2.40
NM_009234	Sox11	SRY-box containing gene 11	−2.25
NM_001081323	Mphosph9	M-phase phosphoprotein 9	−2.25
NM_009906	Tpp1	Tripeptidyl peptidase I	−2.24
NM_054102	Ivns1abp	Influenza virus NS1A binding protein	−2.22
NM_024184	Asf1b	ASF1 Anti-silencing function 1 homolog B (S. cerevisiae)	−2.15
NM_009881	Cdyl	Chromodomain protein, Y chromosome-like	−2.12
NM_008726	Nppb	Natriuretic peptide precursor type B	−2.11
NM_007788	Csnk2a1	Casein kinase 2, alpha 1 polypeptide	−2.04
NM_020618	Smarce1	SWI/SNF related, matrix associated, actin dependent regulator of chromatin, subfamily e, member 1	−2.00
NM_010067	Trdmt1	tRNA aspartic acid methyltransferase 1	−2.00

**Figure 4:**
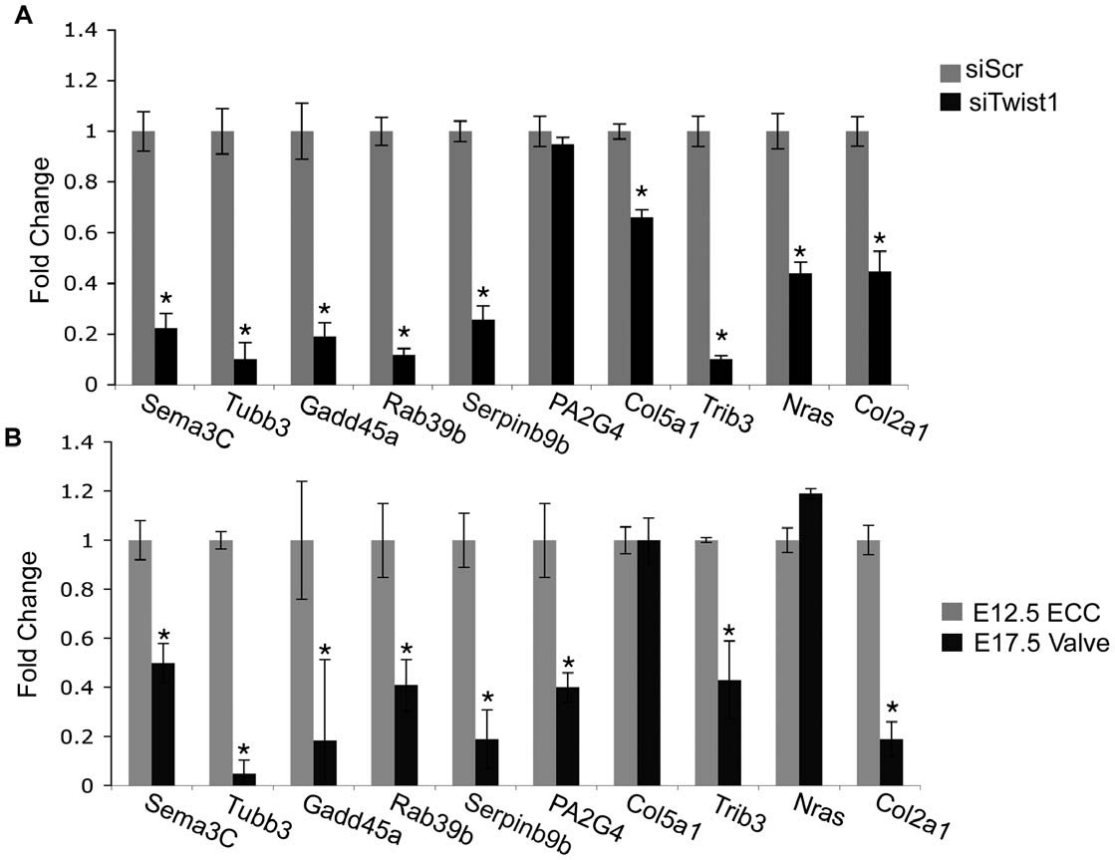
qPCR validation of differential expression of Twist1 candidate target genes in siRNA-transfected MC3T3-E1 cells and in ECC and remodeling valves *in vivo*. A. qPCR was performed to determine the level of expression of candidate Twist1 target genes in MC3T3-E1 cells transfected with siTwist1 versus siScr control. Fold change in expression relative to the level in siScr control cells is shown. B. qPCR was performed to determine the level of expression of Twist1 candidate target genes in mouse E17.5 AV valves versus E12.5 ECCs. Fold change in expression relative to the level in E12.5 ECCs is shown. qPCR determination of the expression of the known Twist1 target gene *Col2a1* was included as a positive control. Statistical significance was determined by Student's *t*-Test, p = ≤0.05 indicated by *. All experiments were performed in biological and technical triplicates. Histograms are a compilation of n = 3 experiments.

Gene expression changes of candidate Twist1 target genes were validated by determination of transcript expression levels of the nine most differentially expressed genes by qPCR of RNA isolated from MC3T3-E1 cells treated with siTwist1 versus siScr. Each of the nine genes chosen for further analysis has been categorized as related to functions regulated by Twist1 (Table 1). Decreased expression of *Sema3c*, *Tubb3*, *Gadd45a*, *Rab39b*, *Serpinb9b*, *Pa2G4*, *Col5a1*, *Trib3*, and *Nras* with loss of Twist1 was confirmed in MC3T3-E1 cells. The differential expression of these Twist1 candidate target genes was validated by qPCR of RNA isolated from MC3T3-E1 cells transfected with siTwist1 or siScr. *Sema3C*, *Tubb3*, *Gadd45a*, *Rab39b*, *Serpinb9b*, *Col5a1*, *Trib3*, and *Nras* expression were significantly decreased in siTwist1 versus siScr treated MC3T3-E1 cells (Figure 4A). Expression analysis of the previously identified Twist1 target gene *Col2a1* was included as a control for differential expression (Figure 4A,B). Differential expression of one candidate gene from the microarray, *Pa2G4*, was not validated in MC3T3-E1 cells. These data indicate that the differential expression of multiple candidate Twist1 target genes identified through microarray gene profiling was validated in MC3T3-E1 cells.

Although a substantial number of genes are expressed in both the developing heart valves and MC3T3-E1 cells, a subset of genes are differentially expressed between preosteoblast cells and heart valve progenitors, that terminally differentiate into distinct cell types [10]. Therefore, expression of candidate Twist1 target genes in the developing heart was examined in vivo. To examine expression of candidate target genes during early versus late heart valve development, qPCR was performed using RNA isolated from mouse E12.5 ECCs, that express high levels of Twist1, compared to RNA isolated from E17.5 AV remodeling valve leaflets, with negligible Twist1 expression (Figure 4A,B). Expression of *Sema3C*, *Tubb3*, *Rab39b*, *Serpinb9b*, *PA2G4*, and *Trib3* were significantly decreased in E17.5 AV valves versus E12.5 ECCs (Figure 4B). Two genes with decreased expression in MC3T3 cells treated with siTwist1, *Col5a1* and *Nras*, showed no differential expression in E17.5 AV valves versus E12.5 ECCs, which may reflect gene regulatory differences in valve progenitor cells and preosteoblast lineages. *Pa2G4* gene expression changes detected by microarray were not confirmed in Twist1 siRNA treated MC3T3-E1 cells, but *Pa2G4* has decreased gene expression in late developing heart valves. These differences could be due to transcript variants of *Pa2G4* differentially detected by microarray and qPCR, or *Pa2G4* may not be directly regulated by Twist1 and thus was not analyzed further. Together these studies demonstrate that multiple candidate Twist1 target genes identified by microarray analysis in MC3T3 cells also are expressed during early heart valve development and are decreased during heart valve remodeling, similar to *Twist1*.

**Figure 5:**
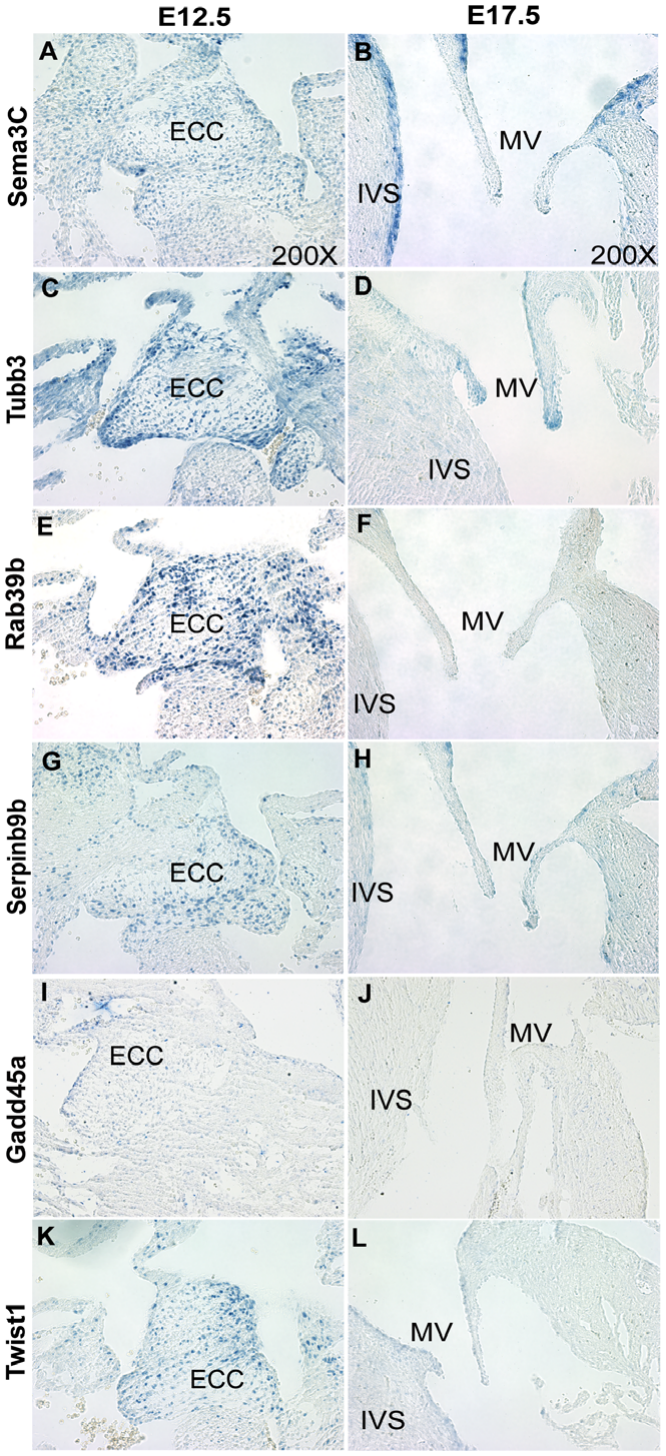
Expression of *Sema3C*, *Tubb3*, *Rab39b*, *Serpinb9b*, and *Gadd45a* is similar to *Twist1* in developing mouse heart valves. *In vivo* localized expression of Twist1 candidate target genes *Sema3C* (A,B), *Tubb3* (C,D), *Rab39b* (E,F), *Serpinb9b* (G,H), and *Gadd45a* (I,J), was determined relative to *Twist1* (K,L) in mouse E12.5 ECCs (A, C, E, G, I, and K) and E17.5 AV valves (B, D, F, H, J, and L) by in situ hybridization of heart sections. The arrows indicate areas of candidate target gene transcript expression. (IVS = interventricular septum, ECC = endocardial cushion, and MV = mitral valve).

To determine whether the candidate Twist1 target genes exhibit expression patterns similar to *Twist1* during heart valve development in E12.5 ECCs and E17.5 remodeling valve leaflets *in vivo*, ISH was performed with the five most differentially expressed genes associated with cell proliferation and migration identified from microarray gene expression profiling (Figure 5). *Sema3C*, *Tubb3*, *Rab39b*, *Serpinb9b*, and *Gadd45a* genes assessed by ISH are responsive to Twist1 in MC3T3-E1 cells and are differentially expressed coincident with Twist1 in mouse valve development as determined by qPCR. Transcript expression and localization of Twist1 candidate target genes, *Sema3C* (Figure 5A,B), *Tubb3* (Figure 5C,D), *Rab39b* (Figure 5E,F), *Serpinb9b* (Figure 5G,H), and *Gadd45a* (Figure 5I,J) is evident in ECCs, that express high levels of *Twist1* (Figure 5K,L). In addition, expression of each is decreased during later valve development (E17.5 mitral valves), when Twist1 expression is low. *Sema3C*, *Tubb3*, *Rab39b*, *Serpinb9b*, and *Gadd45a* also have sparse expression in the interventricular septum (IVS) of the E12.5 heart (Figure 5A,C,E,G,I). Although nearly absent from the E17.5 mitral valves, *Sema3C* and *Serpinb9b* are expressed in the IVS and in AVC myocardium (Figure 5B). Additionally, *Tubb3* retains expression at E17.5 in the distal tips of the mitral valve leaflets (Figure 5D). *Sema3C*, *Serpinb9b*, and *Tubb3* may have Twist1-dependent and independent expression since they exhibit unique and overlapping expression patterns compared to *Twist1* (Figure 5K,L). Thus expression of the Twist1 downstream candidate genes *Sema3C*, *Tubb3*, *Rab39b*, *Gadd45a*, and *Serpinb9b* is similar toTwist1 during heart valve development.

### Twist1 directly binds to ECRs associated with Sema3C, Gadd45a, and Rab39b

**Figure 6:**
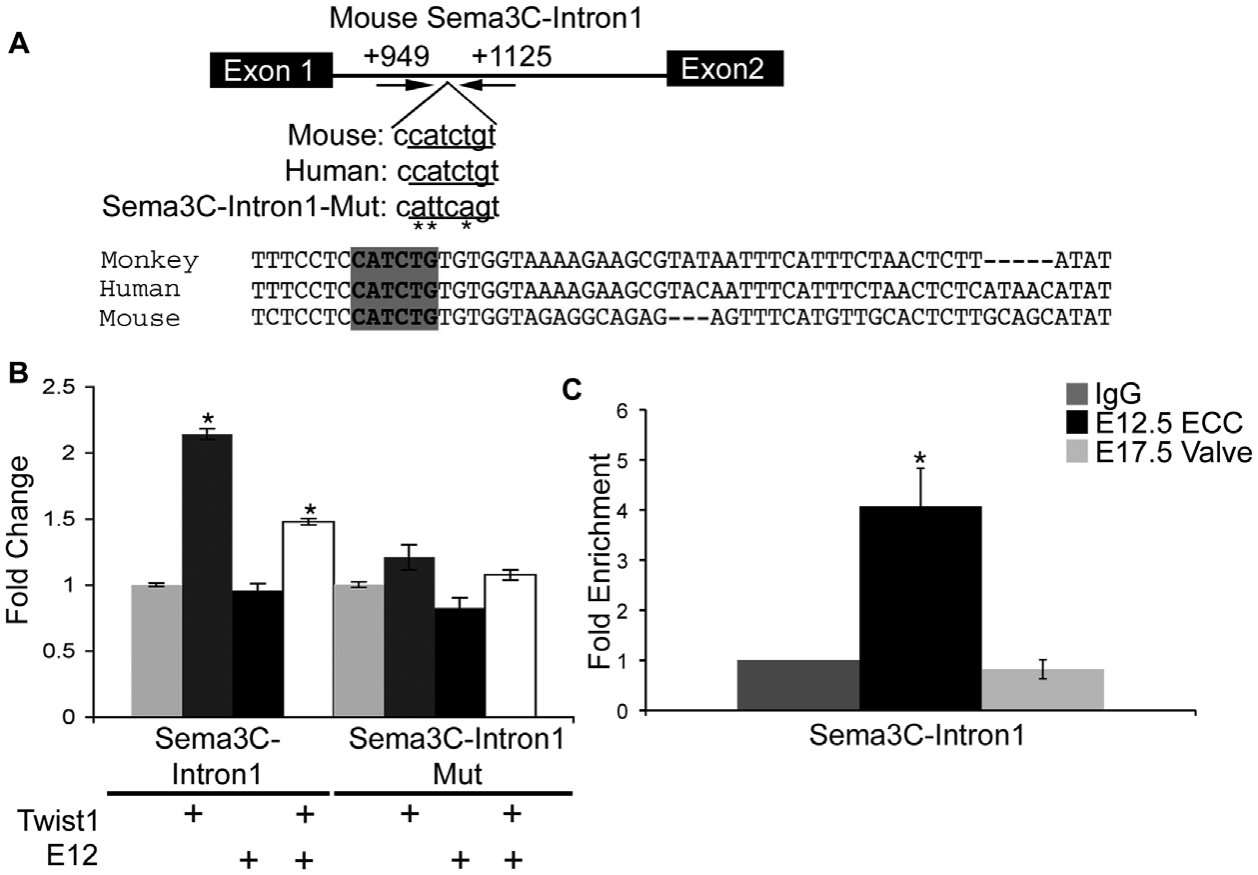
Twist1 binds and promotes gene expression from an ECR within Intron1 of *Semaphorin3C (Sema3C)*. A. An ECR is located +949 to +1125 bps from the mouse *Sema3C* +1 site. Cross-species genomic alignment of the E-box consensus sequence (shaded grey) and mutated sequence (mutated bps indicated by *) are indicated. B. *Sema3C-Intron1* or *Sema3C-Intron1Mut* was co-transfected into HEK293 cells with empty vector or with Twist1, E12, or Twist1 and E12 expression vectors and luciferase reporter assays performed. Fold change over the empty vector control set to 1 is shown with SEM. C. ChIP assays were performed with anti-Twist1 in mouse E12.5 ECCs and E17.5 AV valves and quantified by qPCR. Fold enrichment was evaluated by comparing anti-Twist1 IP of E12.5 ECCs or anti-Twist1 IP of E17.5 valves versus IgG (negative control) set to 1. Statistical significance was determined by Student's *t*-Test, p = ≤0.05 indicated by *. All experiments were performed in biological and technical triplicate. Histograms are a compilation of n = 3 experiments.

DiRe, Trafac, rVista2.0, and oPOSSUM analyses were employed to identify ECRs containing E-box consensus sequences associated with Twist1 candidate target genes identified through microarray gene expression profiling (Table 1). ECRs were selected for further validation based on the presence of an E-box consensus sequence identified by all bioinformatics programs used to analyze genomic alignments and sequence homology between at least mouse and human, since many of the genes identified are not entirely conserved across species. ECRs with these criteria were identified for Twist1 candidate target genes *Sema3C*, *Gadd45a*, and *Rab39b*. *Sema3C* is the gene with the most decreased gene expression (−4.95 fold, Table 1) in the siTwist1 versus siScr microarray gene profiling. An ECR was identified within the 1^st^ intron (+949 to +1125 bps from the +1 site) of *Sema3C* (*Sema3C-Intron1*, Figure 6A). In co-transfection assays to assess enhancer activity, Twist1 promotes reporter gene expression from *Sema3c-Intron1/pGL3p* approximately 2.25-fold. In contrast co-transfection with E12 alone does not result in reporter gene induction compared to *Sema3C-Intron1/pGL3p* co-transfected with the empty vector (Figure 6B). In co-transfection assays of *Sema3C-Intron1/pGL3p* with Twist1 and E12 together, reporter gene expression increases 1.5-fold and is not statistically different from co-transfection with Twist1 alone. To examine whether the E-box consensus sequence in *Sema3C-Intron1* is necessary for Twist1 trans-activation, site-directed mutagenesis was performed to create *Sema3C-Intron1Mut/pGL3p* (CATCTG to ATTCAG, Figure 6A). Co-transfection of *Sema3C-Intron1Mut/pGL3p* with Twist1, E12, or Twist1 and E12 together does not promote reporter gene expression, indicating that Twist1 requires the E-box consensus sequence for induction of gene expression from *Sema3C-Intron1* (Figure 6B). Furthermore, ChIP experiments with anti-Twist1 performed on protein:DNA complexes isolated from ECCs (E12.5 ECCs) and remodeling valves (E17.5 AV valves) confirms that Twist1 directly binds to the *Sema3C-Intron1* sequence preferentially in ECCs *in vivo* (Figure 6C).

**Figure 7:**
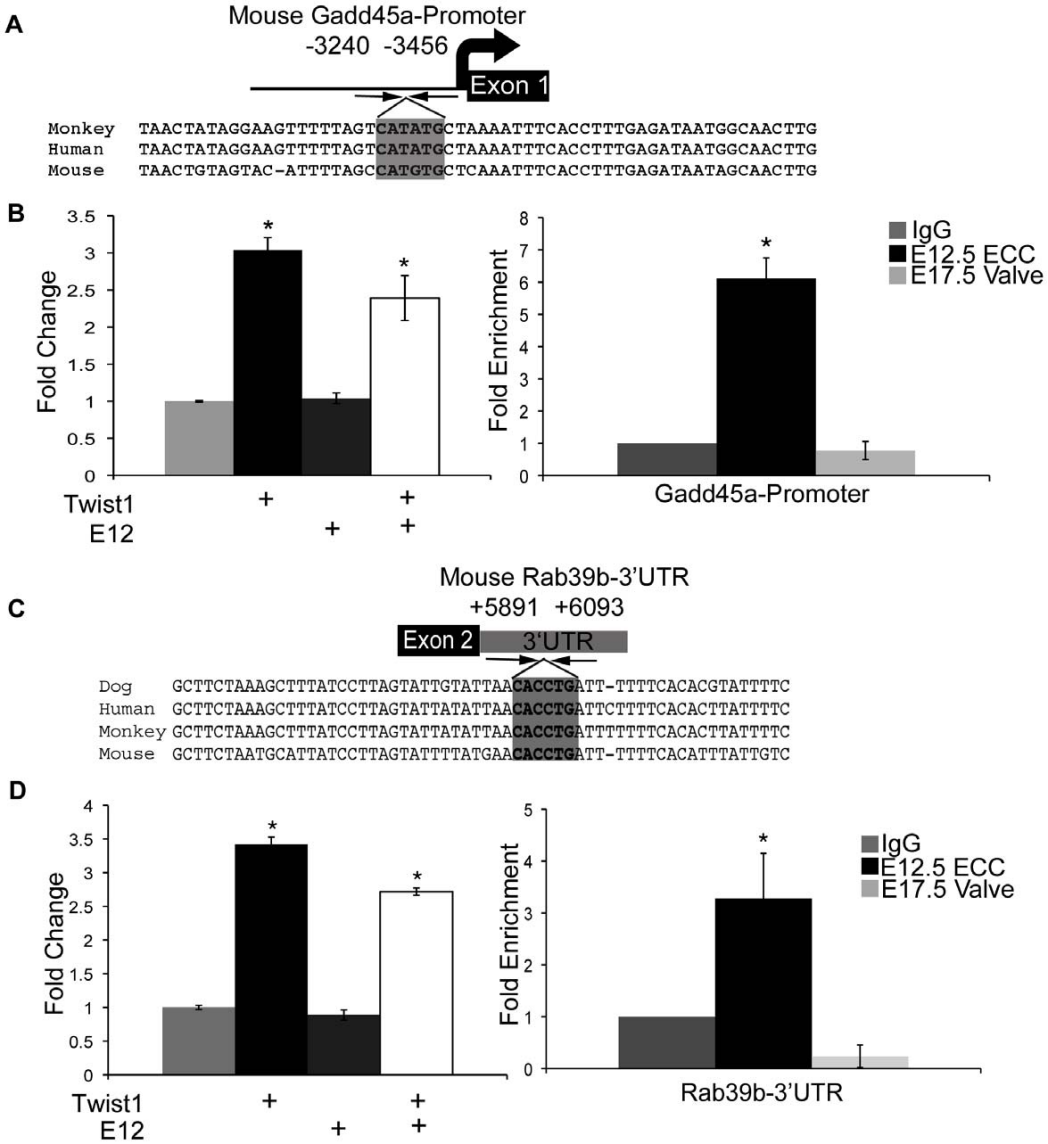
Twist1 binds and promotes gene expression from ECRs associated with *Gadd45a* and *Rab39b*. A. An E-box containing ECR is located −3240 to −3456 bps from the mouse *Gadd45a* +1 site with the E-box sequence indicated by grey shading. B. (left) *Gadd45a*-promoter plasmid was co-transfected into HEK293 cells with empty vector or with Twist1, E12, or Twist1 and E12 expression vectors and luciferase reporter assays performed (left). Fold change over the empty vector control set to 1 is shown with SEM. ChIP assays were performed with anti-Twist1 in mouse E12.5 ECCs and mE17.5 AV valves versus IgG (negative control) set to 1 (right). C. An E-box containing ECR is located within the *mRab39b3′UTR*, +5891 to +6093 bps from *mRab39b* +1 site with the E-box indicated by grey shading. D. *Rab39b-3′UTR* was co-transfected into HEK293 cells with empty vector or with Twist1, E12, or Twist1 and E12 expression vectors and luciferase reporter assays performed (left). Fold change over the empty vector control set to 1 is shown with SEM. ChIP assays were performed with anti-Twist1 in mouse E12.5 ECCs and E17.5 AV valves versus IgG (negative control) set to 1 (right). Statistical significance was determined by Student's *t*-Test, p = ≤0.05 indicated by *. All experiments were performed in biological triplicate. Histograms are a compilation of n = 3 experiments.

The expression level of the DNA repair enzyme-encoding gene *Gadd45a* decreased -4.13 fold in siTwist1 versus siScr treated cells, and an ECR containing an E-box consensus sequences was identified near the 5′-promoter region (*Gadd45a-promoter*, −3240 to −3456 bps from the +1 site, Figure 7A, Table 1). Additionally, expression of the Ras oncogene family member *Rab39b* is significantly decreased by −3.64 fold in siTwist1 versus siScr treated cells, and an E-box containing ECR was identified within the 3′UTR of *Rab39b* (*Rab39b-3′UTR*, +5891 to +6093 bps from the +1 site, Figure 7C, Table 1). Co-transfection assays with *Gadd45a-pr*/pGL3p or *Rab39b-3′UTR*/pGL3p with Twist1 alone or Twist1 and E12 together, but not E12 alone, induced reporter gene expression by approximately 3.25-fold, respectively (Figure 7B,D left panels). Furthermore, ChIP assays on developing heart valves with anti-Twist1 show increased Twist1 binding to *Gadd45a-pr* (6.5-fold enrichment) and *Rab39b-3′UTR* (3.5-fold enrichment) in early E12.5 ECCs relative to later E17.5 AV valves (Figure 7B,D right panels). These data indicate that enhancers associated with Twist1 candidate target genes, *Gadd45a* and *Rab39b*, are responsive to Twist1 in co-transfection assays and are directly bound by Twist1 during heart valve development.

## Discussion

The bHLH transcription factor Twist1 is well established as an essential regulator of mesenchymal cell maintenance, however, identification of Twist1 transcriptional targets remains incomplete. In this study, we report multiple direct Twist1 transcriptional target genes identified through candidate and global gene profiling approaches. These genes all have functions regulating the critical mesenchymal cell characteristics of cell proliferation and migration. Twist1 promotes these cellular functions in various cell types during development, such as developing heart valves, and also in metastatic cancers. Elucidation of Twist1 transcriptional hierarchies regulating cell proliferation and migration will further the understanding of the molecular mechanisms by which Twist1 functions in heart development and cancer progression.

We have identified Twist1-responsive ECRs, predicted to act as gene enhancers, associated with *Tbx20*, *Cdh11*, *Sema3C*, *Gadd45a*, and *Rab39b* genes that promote cell proliferation and migration. These enhancers are directly bound by Twist1 in developing heart valves, and conserved E-box consensus sequences were identified that are required for Twist1-responsive gene expression. Unlike other bHLH transcription factors, whose transcriptional activity requires paired E-box consensus sequences, Twist1 appears to only require one E-box consensus site to promote gene expression [32]. With the exception of *Cdh11*, each of the ECRs identified in this study contains a single E-box consensus sequence. Conversely, *Cdh11-Intron1* contains 2 E-box consensus sequences, however, Twist1 binding and gene induction was detected only for E-box1. rVista2.0, oPOSSUM, DiRE, and Trafac analysis for transcription factor binding sequences revealed that each identified enhancer has additional conserved transcription factor binding consensus sequences [16]–[19]. Enhancer sequences identified in these studies are located in upstream genomic regions, proximal to the gene, in 3′UTR, and intronic gene regions, consistent with locations of previously identified enhancers within the genome [33]. Interestingly, regions within close proximity to the E-box consensus site are enriched for A/T sequences, relative to more distal flanking regions. However, no common binding sequences within close proximity to the E-box consensus site of the Twist1 responsive ECRs were identified (Figures S1, S2, S3, S4). From these data, we predict that Twist1 does not require a specific co-factor protein to promote gene expression from its downstream target genes. Although an obligate Twist1 co-factor was not identified from these experiments, Twist1 binds to the E-box consensus sequence as either a homodimer or heterodimer with E-proteins [11]. In other systems, bHLH dimer composition dictates target gene responsiveness, but dimer-specificity of Twist1 function in heart valve development has not yet been determined [3], [34], [35].

Identified Twist1 target genes involved in cell migration include *Sema3C* and *Cdh11*. *Sema3C* is the gene with the greatest decrease in expression resulting from in siTwist1 treatment of MC3T3-E1 cells (Table 1). Previous studies have demonstrated that Sema3C promotes cell migration of axons, neural crest cells, and metastatic cancer cells [36], [37]. Sema3C null mice die within the first 24 hours of life from persistent truncus arteriosus and aortic arch malformations due to neural crest migration defects [38]. Similar to *Twist1*, *Sema3C* is important for NCC contribution to OFT development, but a role in heart valve development has not previously been reported [38], [39]. The identification of a Twist1-responsive ECR bound by Twist1 in ECCs supports Twist1 activation of *Sema3C* in ECC mesenchymal cells. Whether this hierarchy exists in migrating NCC will require additional investigation. The Twist1-responsive gene, *Cdh11*, also promotes cell migration and has overlapping expression with *Twist1* in preosteoblast cells, neural crest cells, preadipocytes, and heart valve mesenchymal cells [8], [40], [41]. Since *Cdh11* is expressed in multiple migratory cell types, we predict that Twist1 activates *Cdh11* to promote cell migration in ECC mesenchymal cells of the developing heart valves. Additional Twist1 target genes involved in cell migration include *N-cadherin*, identified in cancer cells, and *Zyxin*, identified in ECC mesenchymal cells [13], [15], [42]. Together these studies provide accumulating evidence that Twist1 coordinately regulates multiple downstream target genes to promote cell migration in ECC mesenchymal cells, as well as in other embryonic and cancer cell types.

Twist1 promotes cell proliferation in ECC mesenchymal cells, in addition to other mesenchymal progenitor populations, and also in metastatic cancer cells [5], [11]. Nevertheless, the transcriptional hierarchies by which Twist1 promotes cell proliferation are largely uninvestigated. Our studies identified two direct transcriptional targets of Twist1, *Gadd45a* and *Rab39b*, which have functions in regulation of cell proliferation during embryogenesis and in cultured cells, respectively [43], [44]. However, *Gadd45a* and *Rab39b* expression during heart valve development has not previously been reported. Expression of *Gadd45a* is responsive to multiple cellular stresses, and promotes genomic stability through prevention of DNA damage [28]. Rab39b belongs to the Rab group in the Ras family of small GTPases, which propagate TGF-beta signaling through receptor recycling within the cell inducing cellular proliferation [44]. Additional Twist1 candidate target genes identified by microarray, *Trib3* and *Serpinb9b*, promote proliferation of cancer cells and dendritic cells, respectively [45], [46]. Thus Twist1 promotes cell proliferation in ECC mesenchymal cells, through regulation of multiple genes, including *Gadd45a* and *Rab39b*. It is likely that these regulatory interactions also are important for cell cycle regulation in other mesenchymal cells in the embryo, as well as in cancer cell cycle progression.

During heart valve development, Twist1, along with its target gene Tbx20, regulate the transition from mesenchymal ECC to remodeling valve leaflet [8]. Prolonged expression of Twist1 during heart valve development leads to increased cell proliferation and expression of primitive ECM proteins during heart valve remodeling stages [4]. Additionally, human adult and pediatric diseased aortic valves exhibit increased expression of Twist1, induction of mesenchymal gene expression, increased cell proliferation, and disruption of ECM organization [4], [8], [47]. These characteristics of diseased heart valves are in accordance with Twist1 functions observed in ECC mesenchymal cells. However, it is not clear if these regulatory interactions have reparative or pathologic functions in heart valve disease. It is likely that the same Twist1-activated regulatory hierarchies are important in the development of various cell types, including osteoblast, neural crest cells, and cancer cells. Therefore manipulation of Twist1-mediated regulatory events could be used to develop therapeutic strategies related to both human heart valve disease and cancer progression [5], [48].

## Supporting Information

Figure S1 ***Tbx20boxA*** **ECR cross species genomic alignment and conserved transcription factor binding sites.** The chicken *Tbx20boxA* genomic sequence was utilized for luciferase assays and ChIP assays. Genomic alignment with corresponding zebrafish (NW_001877680.2), rat (NW_047798.2), mouse (NW_001030907.1), and human (NW_001839003.1) conserved sequences is shown. The dashed boxes indicate predicted transcription factor binding sites, and the black lines represent the location of primers used for ChIP assays.
(TIF)

Figure S2 ***Cdh11-Intron1*** **ECR cross species genomic alignment and conserved transcription factor binding sites.** The chicken *Cdh11-Intron1* genomic sequence was utilized for luciferase assays and ChIP assays. Genomic alignment with zebrafish (NW_001879268.3), frog (NW_003163392.1), dog (NW_876316.1), human (NW_0018388290.1), monkey (NW_001111353.1), cow (NW_001493595.2), rat (NW_001084742.1), and mouse (NW_001030904.1) conserved sequences is shown. The dashed boxes indicate predicted transcription factor binding sites, and the black lines represent the location of primers used for ChIP assays.
(TIF)

Figure S3 ***Sema3C-Intron1*** **ECR cross species genomic alignment and conserved transcription factor binding sites.** The mouse *Sema3C-Intron1* genomic sequence was utilized for luciferase assays and ChIP assays. Genomic alignment with human (NW_001839063.1) and monkey (NW_001114280.1) conserved sequences is shown. These sequences were not conserved in zebrafish, frog, chicken, dog, or cow genomes. The dashed boxes indicate predicted transcription factor binding sites, and the black lines represent the location of primers used for ChIP assays.
(TIF)

Figure S4 ***Rab39b-3′UTR*** **, and** ***Gadd45a-prm*** **ECR cross species genomic alignment and conserved transcription factor binding sites.** The mouse *Rab39b-3′UTR* (top) genomic sequence was utilized for luciferase assays and ChIP assays. Genomic alignment to corresponding monkey (NW_001218204.1) and human (NW_001842420.1) conserved sequences is shown. These sequences were not conserved in zebrafish, frog, chicken, dog, or cow genomes. The mouse *Gadd45a-prm* (bottom) genomic sequence was utilized for luciferase assays and ChIP assays. Genomic alignment with monkey (NW_001108704.1) and human (NW_001830579.2) sequences is shown. The dashed boxes indicate predicted transcription factor binding sites, and the black lines represent the location of primers used for ChIP assays.
(TIF)
