# Supplementary figures and images for: Twist1 Directly Regulates Genes That Promote Cell Proliferation and Migration in Developing Heart Valves
Source: PLoS One. 2011 Dec 29;6(12):e29758. doi: 10.1371/journal.pone.0029758 (PMC3248441; doi:10.1371/journal.pone.0029758)

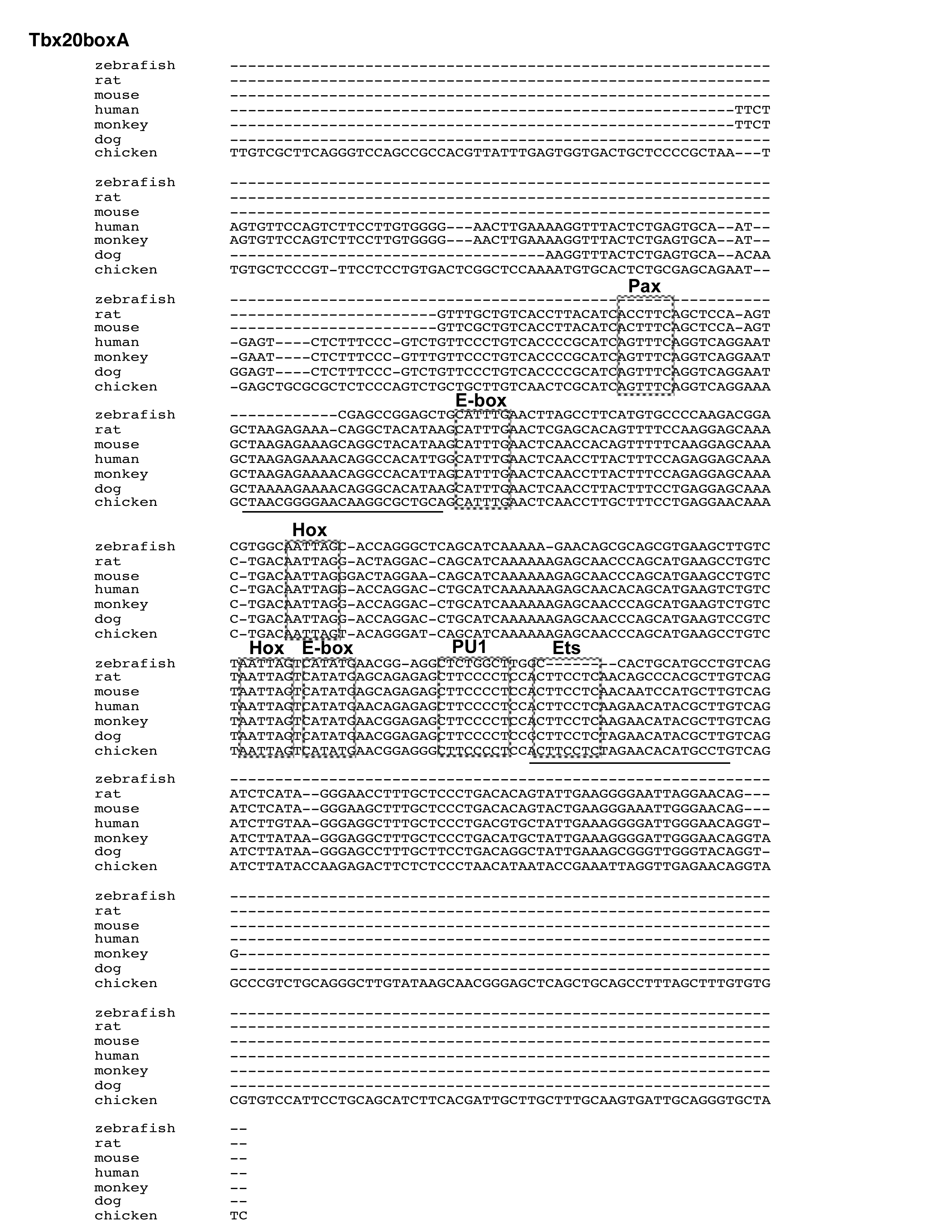

Supplement: Figure S1 — Tbx20boxA ECR cross species genomic alignment and conserved transcription factor binding sites. The chicken Tbx20boxA genomic sequence was utilized for luciferase assays and ChIP assays. Genomic alignment with corresponding zebrafish (NW_001877680.2), rat (NW_047798.2), mouse (NW_001030907.1), and human (NW_001839003.1) conserved sequences is shown. The dashed boxes indicate predicted transcription factor binding sites, and the black lines represent the location of primers used for ChIP assays. (TIF) [file pone.0029758.s001.tif]

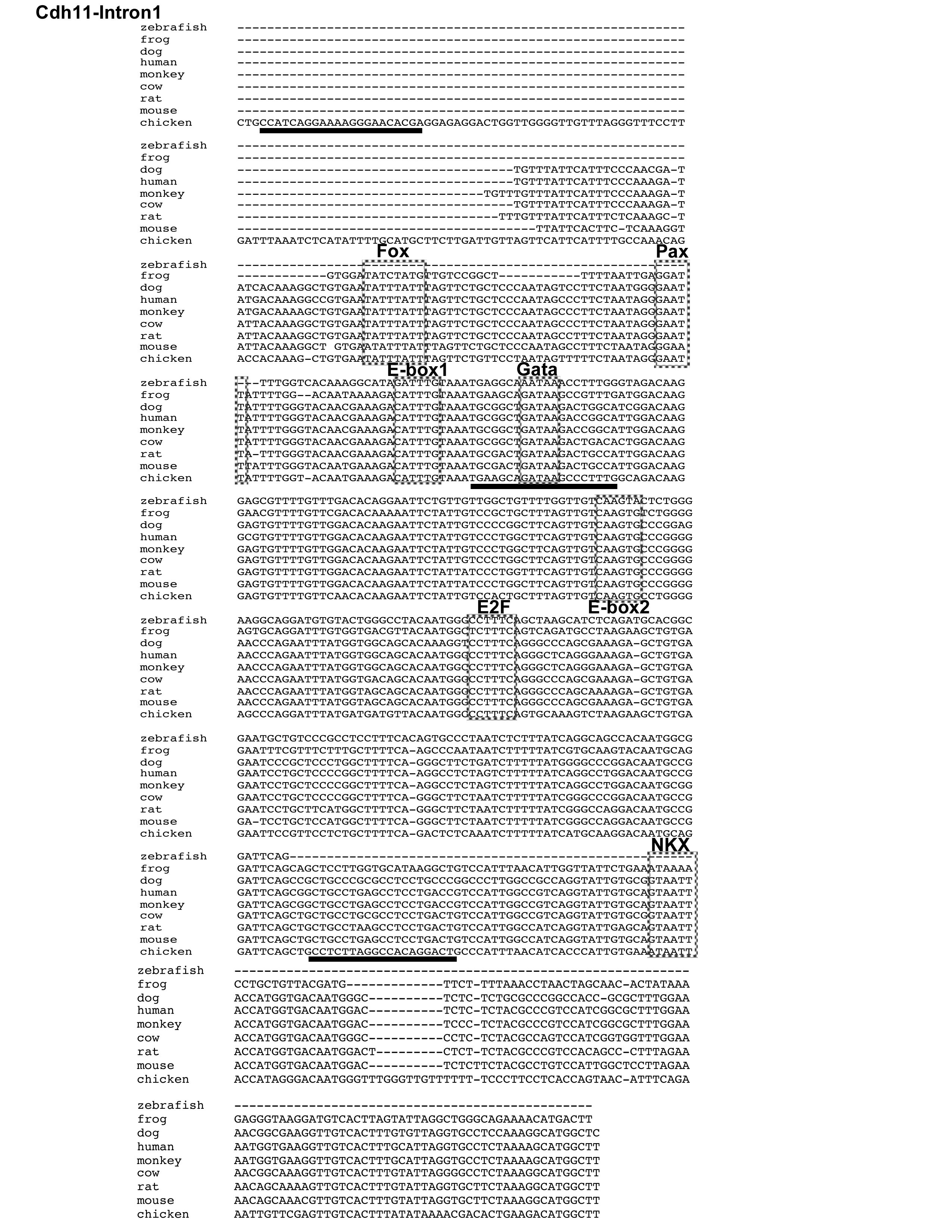

Supplement: Figure S2 — Cdh11-Intron1 ECR cross species genomic alignment and conserved transcription factor binding sites. The chicken Cdh11-Intron1 genomic sequence was utilized for luciferase assays and ChIP assays. Genomic alignment with zebrafish (NW_001879268.3), frog (NW_003163392.1), dog (NW_876316.1), human (NW_0018388290.1), monkey (NW_001111353.1), cow (NW_001493595.2), rat (NW_001084742.1), and mouse (NW_001030904.1) conserved sequences is shown. The dashed boxes indicate predicted transcription factor binding sites, and the black lines represent the location of primers used for ChIP assays. (TIF) [file pone.0029758.s002.tif]

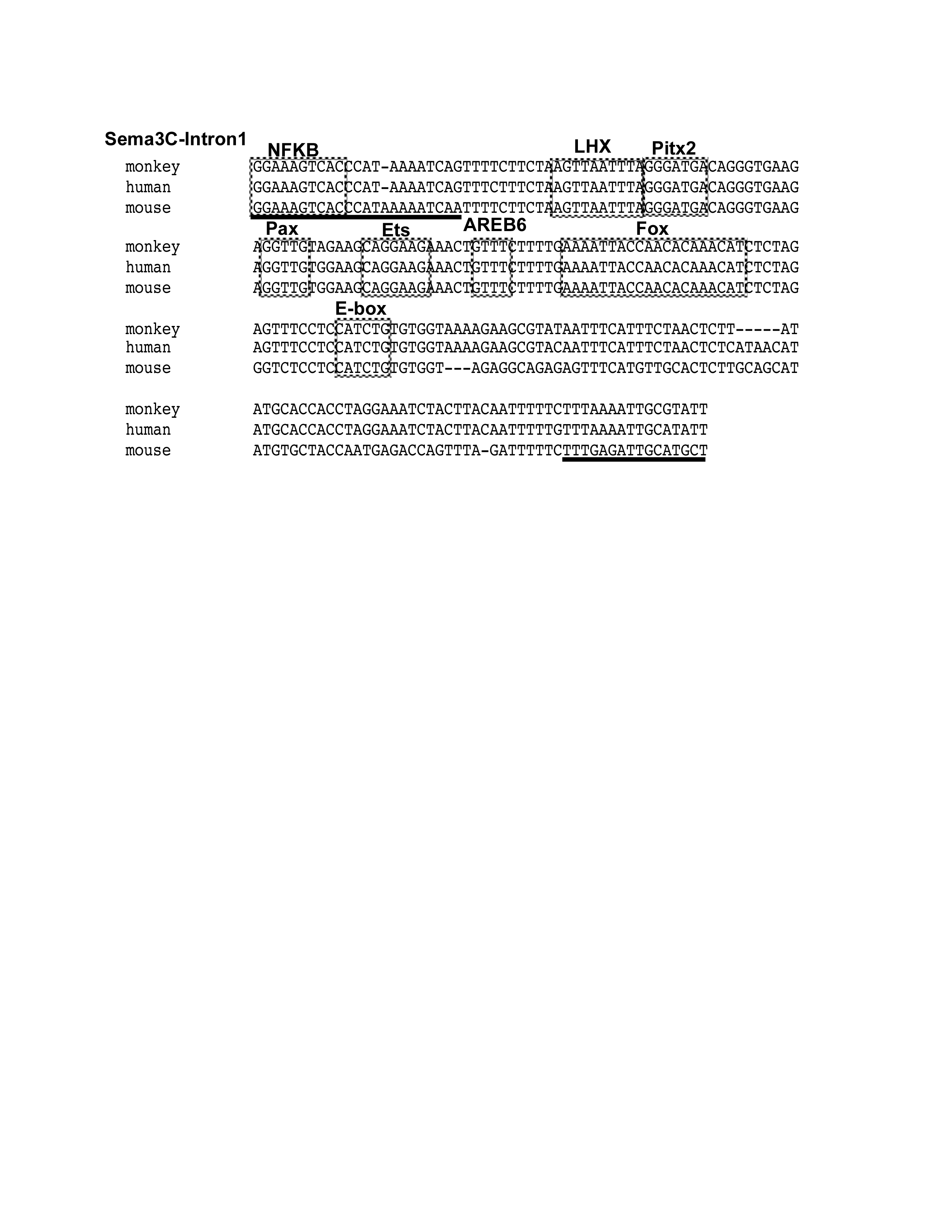

Supplement: Figure S3 — Sema3C-Intron1 ECR cross species genomic alignment and conserved transcription factor binding sites. The mouse Sema3C-Intron1 genomic sequence was utilized for luciferase assays and ChIP assays. Genomic alignment with human (NW_001839063.1) and monkey (NW_001114280.1) conserved sequences is shown. These sequences were not conserved in zebrafish, frog, chicken, dog, or cow genomes. The dashed boxes indicate predicted transcription factor binding sites, and the black lines represent the location of primers used for ChIP assays. (TIF) [file pone.0029758.s003.tif]

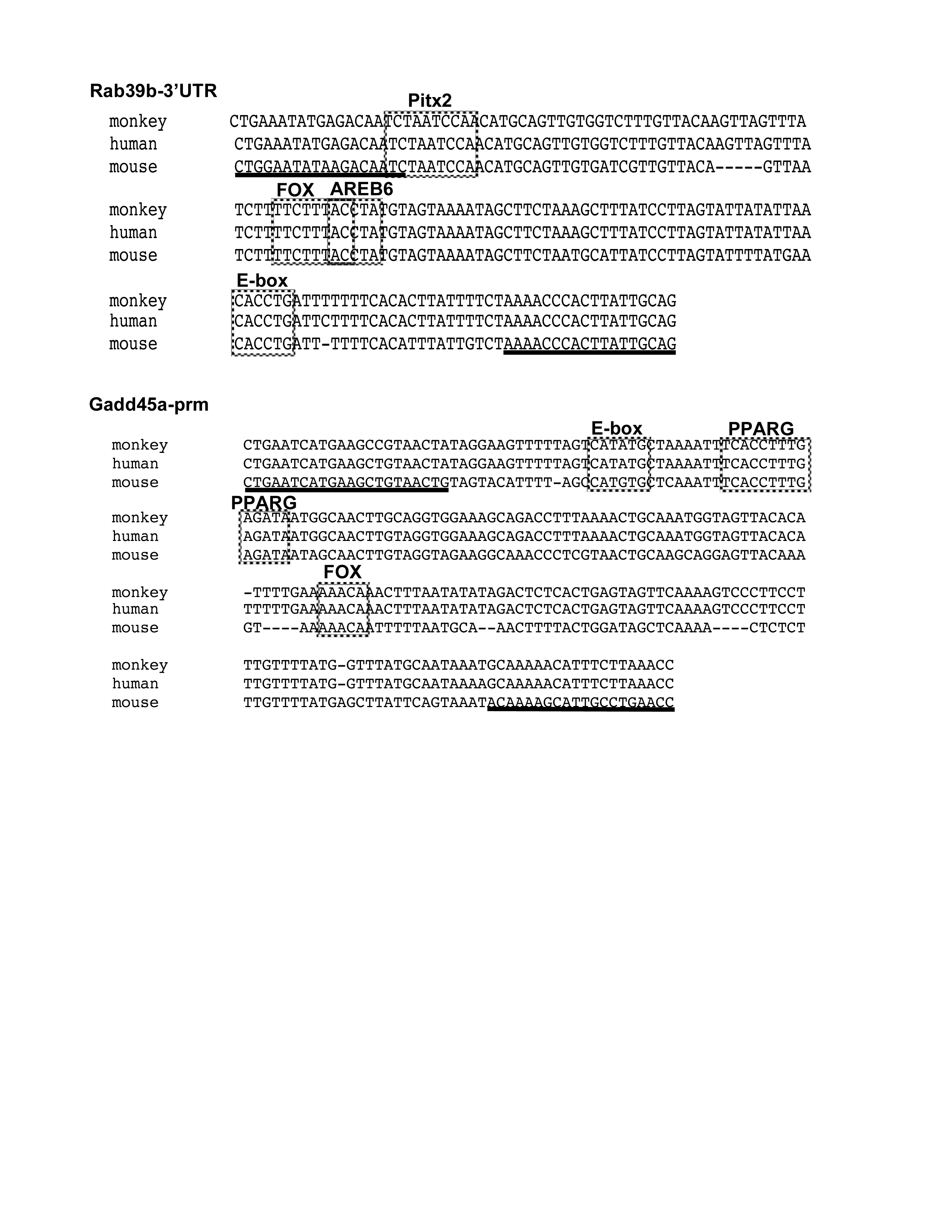

Supplement: Figure S4 — Rab39b-3′UTR , and Gadd45a-prm ECR cross species genomic alignment and conserved transcription factor binding sites. The mouse Rab39b-3′UTR (top) genomic sequence was utilized for luciferase assays and ChIP assays. Genomic alignment to corresponding monkey (NW_001218204.1) and human (NW_001842420.1) conserved sequences is shown. These sequences were not conserved in zebrafish, frog, chicken, dog, or cow genomes. The mouse Gadd45a-prm (bottom) genomic sequence was utilized for luciferase assays and ChIP assays. Genomic alignment with monkey (NW_001108704.1) and human (NW_001830579.2) sequences is shown. The dashed boxes indicate predicted transcription factor binding sites, and the black lines represent the location of primers used for ChIP assays. (TIF) [file pone.0029758.s004.tif]
